# Influence of emotional intelligence on patient-dentist relationships: A questionnaire development, validation and pilot study

**DOI:** 10.1016/j.jobcr.2025.04.003

**Published:** 2025-04-18

**Authors:** J Deepasakthi, Arthi Balasubramaniam, M. Karthik

**Affiliations:** a Department of Public Health Dentistry, Saveetha Dental College & Hospital, Saveetha Institute of Medical and Technical Sciences, Saveetha University, No.162, Poonamallee High Road, Tamil Nadu, Chennai, 600077, India; b Department of Mathematics, Hindustan College of Arts & Science, India

**Keywords:** Emotional intelligence, Dentist-patient relationship, Questionnaire development, Validation, Internal consistency

## Abstract

**Background:**

The Dentist-Patient relationship is a crucial component of successful dental care. Emotional intelligence (EI) has emerged as an important factor that can shape patient experiences and improve the quality of care. The present study was aimed to develop a questionnaire, validate the questionnaire, and assess how EI influences the dentist-patient relationship.

**Materials and methods:**

This study was conducted in four phases. First phase was a focus group discussion with preliminary questionnaire development. Phase II was validation of the preliminary questionnaire and phase III was a pilot study that assessed the internal consistency of the preliminary questionnaire. Phase IV was the survey conducted to assess the influence of EI on dentist-patient relationship using the final questionnaire. A cross-sectional survey was conducted with 200 dentists in the month of September 2024.

**Results:**

The CVI and CVR of the final questionnaire was found to be > 0.8 with κ = 81 % suggestive of good intra-rater agreement. The internal consistency of the final questionnaire was good with Cronbach's α value > 0.7. More than 40 % of the participants had an opinion that emotional intelligence helps in making ethical decisions in dental practice. Also, 41.5 % of the dentists suggested for a formal training in emotional intelligence in dental schools.

**Conclusion:**

By fostering trust, improving communication, and managing emotional challenges, dental professionals can enhance the quality of care and patient experiences. Implementing EI-focused modules into dental curricula can better prepare future dentists to manage the emotional and psychological aspects of patient care.

## Graphical abstract

**Figure:**
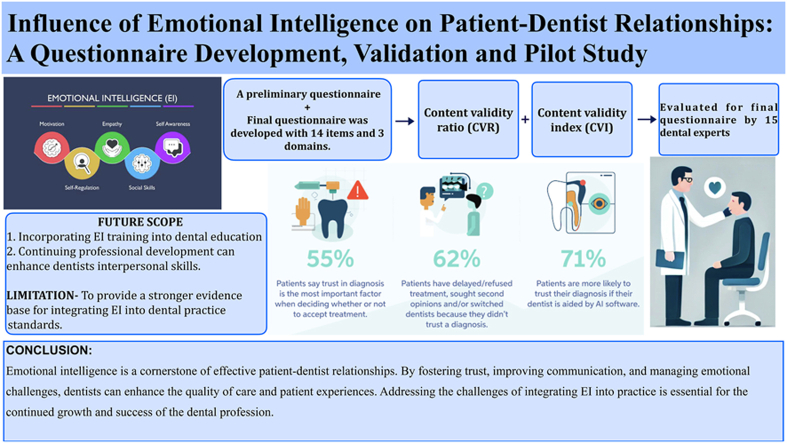

## 1 Introduction

Measuring quality in healthcare programs is essential because it significantly influences healthcare outcomes, costs, and the information available to consumers for making choices. One of the most frequently used indicators of healthcare quality is patient satisfaction.^1^ The patient-dentist relationship is a crucial component of successful dental care, significantly influencing patient outcomes, satisfaction, and adherence to treatment plans. Unlike many other healthcare encounters, dental visits often evoke heightened anxiety and fear, making effective communication and emotional sensitivity essential. Dentists face the unique challenge of managing not only patients’ clinical needs but also their emotional states.^2^ In this context, Emotional Intelligence (EI) has emerged as an important factor that can shape patient experiences and improve the quality of care by demonstrating a positive patient relationship and good rapport.^1^ This conception was introduced by Salovey and Mayer,^3^ expanded by Gardner in the multiple intelligences theory,^4^ and brought into vogue by Goleman in his Emotional Intelligence book.^5^ EI refers to the ability to perceive, understand, and manage emotions, both in oneself and in others. It encompasses skills such as self-awareness, self-regulation, empathy, and effective social interaction, all of which are relevant in fostering trust and comfort in healthcare settings.^6^

In medical and psychological research, high Emotional Intelligence in healthcare professionals has been associated with improved patient communication, reduced anxiety, and greater patient trust.^7^ These attributes are critical in dentistry, where patient cooperation and comfort directly affect the outcome of treatment. For instance, a dentist who can recognize and manage a patient's fear with empathy and reassurance may create a more positive experience, reducing the likelihood of dental phobia and increasing patient satisfaction.^8^ A prosperous dentist-patient relationship helps in increasing patient adherence and loyalty, better anxiety management and treatment results.^9^, ^10^, ^11^ Despite its apparent relevance, the impact of Emotional Intelligence in dentistry remains underexplored in developing countries like India, with most studies focusing on broader medical contexts and in western countries. Understanding how EI shapes patient-dentist interactions is crucial for enhancing patient-centered care in dental practices.^12^

Patient-centered care emphasizes the importance of understanding and addressing patients' individual needs, values, and emotional states. Emotional Intelligence plays a central role in achieving this by enabling dentists to tailor their communication and behavior to each patient's unique concerns.^13^ A high level of EI allows dentists to effectively read patients' emotional cues and adjust their approach accordingly. This can help alleviate fear, make patients feel heard and respected, and foster a trusting environment conducive to better health outcomes. Additionally, EI is essential for dentists in managing their own stress and emotional responses, which can impact the quality of care they deliver.^14^

Although Emotional Intelligence has been widely researched, there is limited information about its specific role in dentistry. Dental undergraduates with higher EI scores have demonstrated a better ability to handle academic and other forms of stress, which contributes to stronger academic performance.^15^ Some evidence also suggests that the EI levels of dentists and dental students improves social interaction and patient satisfaction.^1^^,^^6^^,^^12^ In a dental school setting, achieving high patient satisfaction is particularly important, as administrators must balance fulfilling the needs of both dental students and patients.^15^ Thus, there is a need for the module on development of cognitive, technical, and social skills in dental education.^16^^,^^17^ Health care professionals, educators, and researchers established EI as an important predictor of patient outcomes and clinical performance for students.^18^^,^^19^

Effective communication strategies, good perseverance of emotional states of patients, demonstration of empathy, provision of treatment with structured and professional approach helps in creating a good rapport and therapeutic relationship with patients.^12^ A good dentist-patient relationship considers psychological, pharmacological, interpersonal, communication skills. Thus, there is necessity to study social interactions of dental professionals in a diverse country like India. Although, a tool to assess dentist-patient relationship skills is available in Western countries, there is a lack of instrument centering the dentist, dental practitioner, dental educator defined criteria in India. Methods such as patient perception questionnaires, simulated patients’ perceptions, teacher assessment, self-assessment, self-mentor-patient assessment, and objective structured clinical examinations (OSCEs) are available in assessing EI and communication skills in dental education, these instruments failed to assess dentist-patient interaction focusing on intra operative and postoperative communication which are essential for dental practice in India.^20^ Therefore, the present study addressing the gap, aimed to assess the influence of EI on dentist-patient relationship. The objectives include to propose a professionalism of dentist-patient relationship skills by EI psychological construct from dental fraternities using a focus group discussion, to develop a preliminary face-validated, content, construct validated self-evaluated instrument considering dental practitioner-patient interaction skills, to pilot test for internal consistency the preliminary questionnaire, and to assess the influence of EI on dentist-patient relationship using final questionnaire.

## 2 Materials and methods

### 2.1 Questionnaire development

**Fig. 1:**
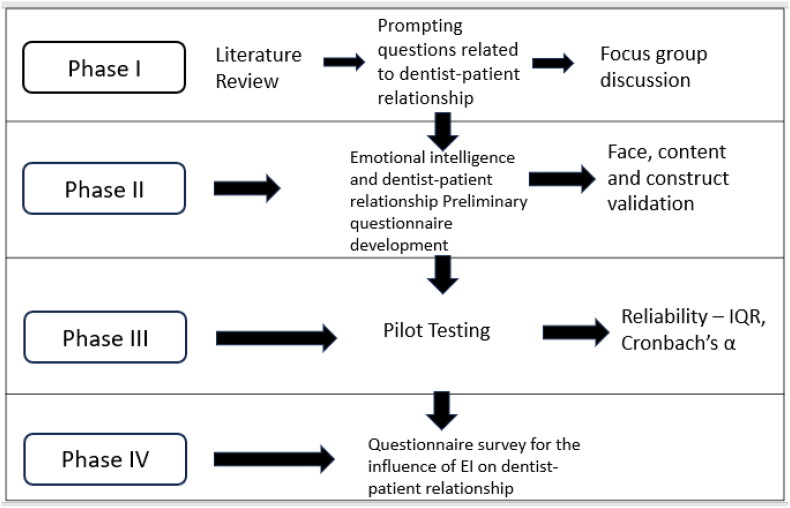
Study Outline in four phases.

The ethical clearance to conduct the study was obtained from the Institutional Review Board of author's institution (SRB/SDC/UG-2010/24/PHD/377). The study was conducted between May and September 2024 in four phases (Fig. 1).

### 2.2 Phase I (focus group discussion)

An audio recorded focus group discussion (FGD)^21^^,^^22^ was carried out in English with five experts, a psychiatrist, a psychologist, a sociologist, a dentist, and an educated patient to develop a self-evaluated questionnaire assessing the influence of emotional intelligence on dentist-patient relationship. The focus group discussion went on for 30 min with prompting questions related to “dental education,” “communication,” “dentist-patient relationship,” “assessment scale,” and “emotional intelligence” identified from a previous study.^13^ The FDG was conducted in authors institution, moderated by the second author. The audio recordings of discussion were then converted to transcripts by an expert not involved in the study. Later, a questionnaire was developed after reading the transcripts for several times by psychologist not involved in FGD and the authors. Questionnaire items with similar assessment were omitted for consistency of the questionnaire. Factor analysis was not performed as the questionnaire items had less set of variables for factor loading. A preliminary questionnaire with 22 items in five domains was developed to assess the influence of emotional intelligence on dentist-patient relationship among the dental practitioners.

### 2.3 Phase II (preliminary questionnaire development, validation)

The preliminary questionnaire from the FGD transcripts with 22 no double barreled, negative question items under five domains was developed (Table 1). This questionnaire was then mailed to 15 dental experts of prosthodontics, endodontics, orthodontics, oral medicine, pedodontics, public health and periodontics having clinical experience not less than five years in Chennai, Tamil Nadu with a cover letter stating the purpose and description of the questionnaire, to rate the items to evaluate face validity, content, and construct validity. Content validity ratio (CVR), a measure of importance of scoring of the questionnaire items (0 – Not necessary; 1-Useful; 2- Essential) was evaluated with the formula.^23^

CVR = (N_E_ –N/2)/(N/2)

where N_E_ = number of experts rated an item as essential; N = Total number of experts.

**Table 1:** Question items in the preliminary assessment.

Domain	Component	Items
Knowledge	knowledge on good dentist-patient relationship	1.1 What are the characteristics of a good dentist-patient relationship?
		1.2 Which elements is lacking to achieve successful dentist-patient relationships in your experience?
		1.3 What is the knowledge required for a dentist to develop a successful rapport with the patient?
		1.4 Is experience needed for a dentist to develop a successful rapport with the patient?
Experience	Necessity of emotional intelligence in clinical practice	2.1 How important do you believe emotional intelligence (EI) in dentistry?
		2.2 How do you typically manage patient emotions (example: anxiety, fear) during dental procedures?
		2.3 How does emotional intelligence contribute to your personal well-being as a dental professional?
Practice	Impact of emotional intelligence in clinical practice	3.1 I am aware when should I share my personal problem with others
		3.2 Other people find it easy to share their issues with me
		3.3 I expect that I will do well on most things I try
		3.4 I am conscious of my emotions when I experience them
		3.5 By looking at their facial expressions, I recognize the emotions of the patients
		3.6 I am conscious of the changes of emotions in me and I have control over them
Opinion	Importance of emotional intelligence in clinical practice	4.1 Emotional intelligence is essential for effective patient communication
		4.2 Emotional intelligence helps dentists manage patient anxiety and fear during procedures
		4.3 Emotional intelligence enhances teamwork and collaboration within dental practices
		4.4 Formal training in emotional intelligence is beneficial for dental students
		4.5 Emotional intelligence contributes to ethical decision-making in dental practice
Belief	Self-belief on emotional intelligence	5.1 I believe that good things will happen
		5.2 I believe some activities will make me happy
		5.3 New ideas will come up when I am in a positive Mood
		5.4 Solving problems is easy when I am in a positive mood

Also, content validity index (CVI) to measure the representativeness (1 – Not representative; 2- Need major revisions to be representative; 3- Need minor revisions to be representative) and clarity (1- Not clear; 2- Need major revision to be clear; 3- Need minor revisions to be clear; 4- Clear) of scoring was evaluated. The following formulas were used to calculate CVI^23^

CVI (Representative) = N_R_ /N

where N_R_ = number of experts rated an item as representative; N = Total number of experts

CVI (Clarity) = Nc /N

where Nc = number of experts rated an item as clear; N = Total number of experts.

Question item with CVR and CVI <0.8 was considered for rewording, rephrasing, and reordering, which was sent again for evaluation to the same experts for the improvement of validation index scores. The inter-rater agreement for the construction of questionnaire was evaluated using Kappa statistics.^23^

### 2.4 Phase III (pilot testing for reliability)

The preliminary questionnaire was then subjected for inter-question correlation (IQC) and internal consistency evaluation using Cronbach's α reliability test from 50 dental practitioners in Chennai. Tamil Nadu. The question items with IQC <0.3 and Cronbach's α < 0.7 were considered for elimination. Question items with IQC <0.3 were deleted to check for the improvement of Cronbach's α value of >0.7. On eliminating the questions with IQR <0.3, a final questionnaire with 14 question items under three domains were developed with considerable internal consistency Cronbach's α value > 0.7.

### 2.5 Phase IV (cross-sectional survey)

The self-administered pre-validated questionnaire (Table 2) was given to 200 dentists in the month of November to December 2024 in Chennai, Tamil Nadu. A convenience sampling technique was used to recruit the participants into the cross-sectional survey. The sample size was calculated using the mean EI scores from the previous study,^1^ with α error of 5 % and power of 80 %. Practicing dentists in urban and semi-urban areas with 5–10 years of experience were recruited. As a buffer to increase the response rate, the questionnaire was sent to 250 dental practitioners in Chennai. Data from participants used for reliability testing (pilot study) were not included for the survey. After obtaining virtual written consent from the dentists, the questionnaire was mailed to each of them as a google forms to the participants. The mail ID of the participants were obtained from the Indian Dental Association (IDA), Chennai branch after explaining the purpose of the study. The response time lasted from 0 h to 7 days. A regular follow-up of the non-respondents was made and a reminder mail was sent for early response. Since we had a buffer of 50 responses, we received 200 responses which was 100 %.

**Table 2:** Final questionnaire with domains and items.

Domain	Question item	Response item				
**Experience**	How important do you believe emotional intelligence (EI) in dentistry?	Not important at all	Somewhat important	Moderately important	Very important	**-**
	How do you typically manage patient emotions (example: anxiety, fear) during dental procedures?	Comforting words & reassurance	Distraction techniques (example: music)	Empathetic listening & understanding	Sedation options	**-**
	How does emotional intelligence contribute to your personal well-being as a dental professional?	Enhances resilience to stress & burnout	Promotes work-life balance	Supports self-awareness & self-regulation	**-**	**-**
**Practice**	I am aware when should I share my personal problem with others	Strongly disagree	Disagree	Neutral	Agree	Strongly agree
	Other people find it easy to share their issues with me	Strongly disagree	Disagree	Neutral	Agree	Strongly agree
	I expect that I will do well on most things I try	Strongly disagree	Disagree	Neutral	Agree	Strongly agree
	I am conscious of my emotions when I experience them	Strongly disagree	Disagree	Neutral	Agree	Strongly agree
	I am conscious of the changes of emotions in me and I have control over them	Strongly disagree	Disagree	Neutral	Agree	Strongly agree
	By looking at their facial expressions, I recognize the emotions of the patients	Strongly disagree	Disagree	Neutral	Agree	Strongly agree
**Opinion**	Emotional intelligence is essential for effective patient communication	Strongly disagree	Disagree	Neutral	Agree	Strongly agree
	Emotional intelligence helps dentists manage patient anxiety and fear during procedures	Strongly disagree	Disagree	Neutral	Agree	Strongly agree
	Emotional intelligence enhances teamwork and collaboration within dental practices	Strongly disagree	Disagree	Neutral	Agree	Strongly agree
	Formal training in emotional intelligence is beneficial for dental students	Strongly disagree	Disagree	Neutral	Agree	Strongly agree
	Emotional intelligence contributes to ethical decision-making in dental practice	Strongly disagree	Disagree	Neutral	Agree	Strongly agree

### 2.6 Statistical analysis

Content validity ratio (CVR) and content validity index (CVI) was calculated in Microsoft Excel using the above-mentioned formula. Inter-rater agreement for the construct validity of the questionnaire was calculated using Kappa statistics in Statistical package for the Social Sciences (SPSS; IBM, Chicago) Version 23.0. Inter-question relationship and Cronbach's α was calculated in SPPS version 23.0. The demographic details of the participants and the responses to the questionnaire items were presented as frequency percentage. The responses among the male and female participants were compared using Chi-square test. A p value < 0.05 was considered significant.

## 3 Results

### 3.1 Validation

a) Content validity ratio (CVR)

Three question items of the preliminary questionnaire had a CVR value −1 were rephrased, five questions which had CVR value < 0.8 were reworded (Table 3).

b) Content validity index (CVI)

**Table 3:** Content validity assessment of the question items in the questionnaire.

Domain	Question	Content Validity Ratio (CVR)	Content Validity Index – Representativeness (CVI-R)	Content Validity Index – Clarity (CVI-C)
Domain 1	Q1	0.81	0.95	0.81
	Q2	0.88	1.00	0.84
	Q3	0.87	0.69	0.86
Domain 2	Q1	0.90	0.94	0.87
	Q2	0.74	0.91	0.85
	Q3	0.82	1.00	0.82
	Q4	0.89	0.00	0.92
	Q5	−0.48	0.00	0.00
	Q6	0.91	0.94	0.84
Domain 3	Q1	0.91	0.95	0.88
	Q2	0.54	0.91	0.87
	Q3	0.79	0.81	0.93
	Q4	0.95	0.76	0.85
	Q5	0.87	0.63	0.88
	Q6	−0.64	0.00	0.00
Domain 4	Q1	0.78	0.40	0.89
	Q2	0.86	0.92	0.81
	Q3	0.61	0.52	0.72
	Q4	0.59	0.00	0.00
Domain 5	Q1	0.83	1.00	0.92
	Q2	0.53	0.49	0.58
	Q3	−0.65	0.00	0.57

Content validity index was measured in two entity such as representativeness and clarity. CVI- representativeness (CVI-R) score of question items <0.8 were revised. Five question items that had CVI-R score zero were reconstructed. Similarly, CVI – clarity (CVI-C) score of question items <0.8 were revised. Three questions which had zero score reconstructed with the opinion of the experts (Table 3).

c) Inter-rater agreement (IRA)

The inter-rater agreement for the construct criterion of the questionnaire measured using Kappa statistics had a significant 81 % agreement between the raters. The CVR and CVI measures does not account for the chance factor whereas IRA accounts for the chance factor (Table 4).

**Table 4:** Inter-rater agreement for the construct validity of the questionnaire.

Agreement	Kappa Value	p-value
Inter-rater	0.812	0.002

### 3.2 Reliability

#### 3.2.1 Inter-question correlation (IQC) and Cronbach's α

**Fig. 2:**
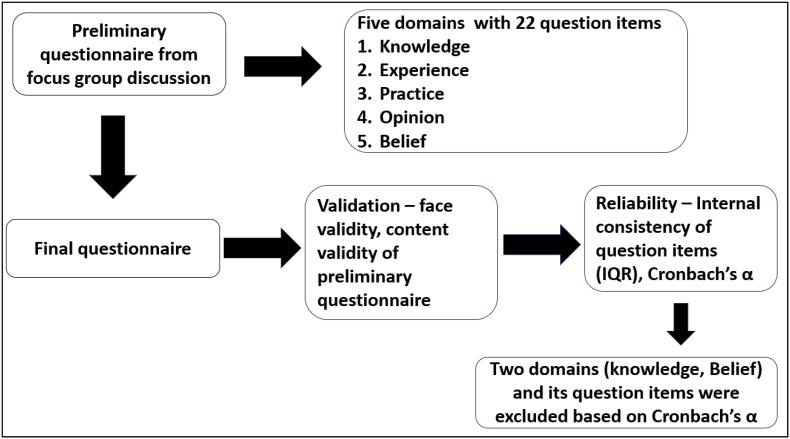
Step-wise arrival of the final questionnaire.

Three questions which had IQC value in minus were eliminated first and five question items with IQC <0.3 were eliminated then. The Cronbach's α from 0.568 improved to 0.742 with 14 question items having Cronbach's α value < 0.7 (Table 5, Fig. 2).

**Table 5:** Internal consistency (reliability) of the question items in the questionnaire.

Question	Inter-question correlation	Cronbach's α if item deleted
Q1	0.423	0.568
Q2	0.413	0.570
Q3	0.394	0.576
Q4	0.334	0.614
Q5	0.392	0.575
Q6	0.430	0.573
Q7	0.158	0.714
Q8	0.308	0.594
Q9	0.546	0.533
Q10	0.543	0.648
Q11	−0.150	0.725
Q12	0.487	0.498
Q13	0.514	0.368
Q14	0.357	0.451
Q15	0.280	0.728
Q16	−0.184	0.730
Q17	0.384	0.439
Q18	0.234	0.731
Q19	0.215	0.733
Q20	0.549	0.465
Q21	−0.138	0.739
Q22	0.249	0.742

#### 3.2.2 Cross-sectional survey

The demographic details of the participants were presented in Table 6. The mean age of the participants was 28.7 years, with male: female ratio of 2:1. Most of the practicing dentists (67.4 %) were from urban areas with an undergraduate degree (62.1 %). About 33.3 % of them felt that emotional intelligence is moderately important in dentistry; 37.3 % manage their patient's emotion (example: anxiety, fear) by comforting words and reassurance. Also, 40 % of the dentists felt that emotional intelligence will improve their self-awareness and self-regulation. Among the dentists, 37.5 % were confident that they will recognize the emotions of the patients by looking at their face; 37.3 % of them are aware their emotional changes happening in them and have a control over them. Greater than 40 % of the participants had an opinion that emotional intelligence not only helps to manage patients' emotion during dental procedures, also helps in making ethical decisions in dental practice (Table 7). No significant difference in the response of items in experience, practice and opinion domain was elucidated between male, female; urban, semi-urban; and undergraduate, postgraduate dentists (p > 0.05).

**Table 6:** Demographic details of the respondents in phase IV.

Demographic details	Measure
Age in years	**Mean ± SD; range**
	28.7 ± 3.256 years; 25–30 years
**Gender**	**N (%)**
Male	150 (75 %)
Female	50 (25 %)
**Location of Dental Practice**	
Urban	135 (67.5 %)
Semi-Urban	65 (32.5 %)
**Highest Degree Obtained**	
Undergraduate	124 (62 %)
Postgraduate	76 (38 %)

**Table 7:** Influence of emotional intelligence on dentist-patient relationship.

Domain	N (%)				
**Experience**					
**Question: 1**	Not important at all	Somewhat important	Moderately important	Very important	
How important do you believe emotional intelligence (EI) in dentistry?	19 (9.3)	56 (28)	66 (33.2)	59 (29.5)	
**Question: 2**	Comforting words & reassurance	Distraction techniques (example: music)	Empathetic listening & understanding	Sedation options	
How do you typically manage patient emotions (example: anxiety, fear) during dental procedures?	75 (37.3)	37 (18.7)	56 (28)	32 (16)	
**Question: 3**	Enhances resilience to stress & burnout	Promotes work-life balance	Supports self-awareness & self-regulation		
How does emotional intelligence contribute to your personal well-being as a dental professional?	56 (28)	64 (32)	80 (40)		
**Practice**					
**Questions**	Strongly disagree	Disagree	Neutral	Agree	Strongly agree
I am aware when should I share my personal problem with others	29 (14.5)	27 (13.5)	51 (25.5)	64 (32)	29 (14.5)
Other people find it easy to share their issues with me	13 (6.3)	32 (16)	48 (24)	85 (42.7)	22 (11)
I expect that I will do well on most things I try	26 (13)	48 (24)	82 (41)	27 (13.5)	14 (7)
I am conscious of my emotions as I experience them	13 (6.3)	24 (12)	51 (25.5)	75 (37.3)	37 (18.5)
I am conscious of the changes of emotions in me and I have control over them	19 (9.5)	32 (16)	53 (26.5)	72 (36)	24 (12)
By looking at their facial expressions, I recognize the emotions of the patients	17 (8.5)	18 (9)	42 (21)	48 (24)	75 (37.5)
**Opinion**					
Emotional intelligence is essential for effective patient communication	16 (8)	16 (8)	40 (20)	99 (49.5)	29 (14.5)
Emotional intelligence helps dentists manage patient anxiety and fear during procedures	16 (8)	24 (12)	35 (17.5)	83 (41.5)	42 (21)
Emotional intelligence enhances teamwork and collaboration within dental practices	16 (8)	22 (11)	37 (18.5)	46 (23)	79 (39.5)
Formal training in emotional intelligence is beneficial for dental professionals	17 (8.5)	17 (8.5)	42 (21)	41 (20.5)	83 (41.5)
Emotional intelligence contributes to ethical decision-making in dental practice	20 (10)	20 (10)	40 (20)	40 (20)	80 (40)

## 4 Discussion

Emotional intelligence (EI) has emerged as a vital factor influencing the quality and success of patient-dentist relationships. This discussion explores the practical implications of EI in dental practice, its potential benefits, and challenges in fostering emotional competence among dental professionals. Patients often experience anxiety or fear during dental visits, which can hinder their cooperation and adherence to treatment plans.^24^ Dentists with strong EI are better equipped to recognize and address empathy which actively acknowledging patient's concerns fosters a sense of safety and trust.^25^ For example, a dentist who listens attentively and provides reassurance can ease a patient's apprehension.^25^ Nonverbal Communication is maintaining eye contact, offering a calming tone, and using open body language to enhance rapport.^26^ By establishing an emotionally supportive environment, dentists can reduce patient anxiety and build lasting professional relationships.^27^

One study that assessed the influence of EI on the stress among the dental undergraduates reported an inverse relationship in contrast to the present study.^6^ One other study which found that dental students with high EI were able to manage the patient's anxiety, identify the ethical issues and recognize the patient's psychosocial issues.^12^ The qualities had led to accurate diagnosis and effective treatment processes.^12^ The present study also confirms that dental professionals with high EI had good dentist-patient relationship.

A previous study which developed an instrument to define the dentist-patient relationship skills had a good internal consistency of Cronbach's α value of 0.95.^13^ This instrument has not been tested for validity, reliability, and transference. However, principal component analysis (PCA) with oblique rotation was conducted for factor reduction and justified for four and six component in social and basic psychological dimensions.^13^ The present study also developed a questionnaire for Indian context which was valid and reliable but no factor analysis conducted, since the questionnaire items had less set of variables for factor loading.

The same previous study after the development of the instrument, conducted a pilot study among 444 dental students of 3rd, 4th, and 5th year students.^13^ The study results in consistency with the present study reported no significant difference in the dentist-patient relationship skills between males and females; and between the year of the study.^13^ Another study that examined the emotional intelligence and patient satisfaction among dental students in consistency with the results of the present study showed that patients of the students with high EI were more satisfied with treatment than the patients of the students with low EI.^1^ A study which compared the personality as a measure for professional behavior in a dental school, found that students with similar personality to dental practitioners performed better in their first year of coursework.^28^ The present study suggest that irrespective of their highest obtained degree and location of their practice, dental professionals with good psychosocial behavior had good dentist-patient relationship.

Effective communication is central to delivering quality care. Adjusting their communication style to suit individual patient needs, whether addressing a child's fear or explaining complex procedures to an adult. Encouraging patients to voice their concerns and preferences, which can lead to better-informed decision-making.^29^ Dental practice often involves high-pressure situations, such as managing patient dissatisfaction or handling emergencies. EI helps dentists by remaining calm and using conflict resolution skills, they can de-escalate tense interactions. Recognizing their emotional triggers allows dentists to maintain professionalism and provide consistent care. This ability to manage emotions not only improves patient relationships but also prevents burnout among dental professionals.^30^

Despite its benefits, integrating EI into dental practice, many dental education programs focus heavily on technical skills, with limited emphasis on emotional and interpersonal development. The fast-paced nature of dental practice may leave little time for emotional engagement with patients. Sensitivity to diverse cultural backgrounds and expectations requires tailored emotional approaches, which can be difficult to navigate without proper training.^31^

The strength of the present study is the development of a questionnaire to assess the influence of EI on dentist-patient relationship among the dentist in India. Also, the questionnaire was validated with good content, construct criteria and reliability measure. Apart from that, this study conducted a survey among the 200 practicing dentists not less than five years of experience from various specialty. The limitation of the study is that failed to assess more social and psychological skill dimensions. Also, failed to the assess the EI quantitatively and find its correlation with dentist-patient relationship. Future studies could investigate the long-term effects of high EI among dentists on patient outcomes, such as reduced dental anxiety, improved treatment adherence, and better oral health. Incorporating EI-focused modules into dental curricula can better prepare future dentists to manage the emotional and psychological aspects of patient care. Investigating how AI-driven tools (e.g., chatbots for patient engagement) can complement dentists’ emotional intelligence could lead to innovative patient communication solutions.^32^

## 5 Conclusion

The preliminary questionnaire developed after focus group discussion with 22 question items, 5 domain which had a content validity ratio and content validity index >0.8 with good inter-rater agreement of κ of 0.81 %. The final questionnaire with 3 domains and 14 question items had good internal consistency and inter-question correlation of >0.7 and > 0.3. More than 50 % of the practicing dentist felt that emotional intelligence is important in dentistry which will improve their self-awareness, self-regulation and helps in making ethical decisions in their practice. Emotional intelligence is a cornerstone of effective patient-dentist relationships. By fostering trust, improving communication, and managing emotional challenges, dentists can enhance the quality of care and patient experiences. Addressing the challenges of integrating EI into practice is essential for the continued growth and success of the dental profession. Also, it has been suggested for integrating emotional intelligence into dental education.

## Funding

No Funding

## Declaration of competing interest

All the authors declare that there was no conflict of interest in the present study.
